# COVID-19 at a Glance: An Up-to-Date Overview on Variants, Drug Design and Therapies

**DOI:** 10.3390/v14030573

**Published:** 2022-03-10

**Authors:** Domenico Iacopetta, Jessica Ceramella, Alessia Catalano, Carmela Saturnino, Michele Pellegrino, Annaluisa Mariconda, Pasquale Longo, Maria Stefania Sinicropi, Stefano Aquaro

**Affiliations:** 1 Department of Pharmacy, Health and Nutritional Sciences, University of Calabria, 87036 Arcavacata di Rende, Italy; domenico.iacopetta@unical.it (D.I.); jessica.ceramella@unical.it (J.C.); michele.pellegrino@unical.it (M.P.); s.sinicropi@unical.it (M.S.S.); stefano.aquaro@unical.it (S.A.); 2 Department of Pharmacy-Drug Sciences, University of Bari “Aldo Moro”, 70126 Bari, Italy; 3 Department of Science, University of Basilicata, 85100 Potenza, Italy; carmela.saturnino@unibas.it (C.S.); annaluisa.mariconda@unibas.it (A.M.); 4 Department of Chemistry and Biology, University of Salerno, Via Giovanni Paolo II, 132, 84084 Fisciano, Italy; plongo@unisa.it

**Keywords:** COVID-19, repositioning, repurposing, pandemic, SARS-CoV-2, vaccines, therapies, drugs, nutraceuticals

## Abstract

Severe acute respiratory syndrome coronavirus-2 (SARS-CoV-2) is a member of the Coronavirus family which caused the worldwide pandemic of human respiratory illness coronavirus disease 2019 (COVID-19). Presumably emerging at the end of 2019, it poses a severe threat to public health and safety, with a high incidence of transmission, predominately through aerosols and/or direct contact with infected surfaces. In 2020, the search for vaccines began, leading to the obtaining of, to date, about twenty COVID-19 vaccines approved for use in at least one country. However, COVID-19 continues to spread and new genetic mutations and variants have been discovered, requiring pharmacological treatments. The most common therapies for COVID-19 are represented by antiviral and antimalarial agents, antibiotics, immunomodulators, angiotensin II receptor blockers, bradykinin B2 receptor antagonists and corticosteroids. In addition, nutraceuticals, vitamins D and C, omega-3 fatty acids and probiotics are under study. Finally, drug repositioning, which concerns the investigation of existing drugs for new therapeutic target indications, has been widely proposed in the literature for COVID-19 therapies. Considering the importance of this ongoing global public health emergency, this review aims to offer a synthetic up-to-date overview regarding diagnoses, variants and vaccines for COVID-19, with particular attention paid to the adopted treatments.

## 1. Introduction

Coronavirus disease 2019 (COVID-19), the highly contagious infectious disease caused by severe acute respiratory syndrome coronavirus 2 (SARS-CoV-2), has had a catastrophic effect on the world’s demographics, resulting in more than 5.8 million deaths worldwide and emerging as the most consequential global health crisis since the era of the influenza pandemic of 1918 [1]. The outbreak of this novel disease has become a global health emergency and continues to rapidly spread around the world. Up to 15 February 2022, more than 410,000,000 cases with COVID-19 have been diagnosed and about 5800,000 death cases have been recorded [2]. Italy was “hit by nothing short of a tsunami of unprecedented forces, punctuated by an incessant stream of deaths. [...] Italy’s biggest crisis since World War II” [3]. Vaccination has been a very effective public health intervention, and different types of vaccines are used worldwide. As of 14 February 2022, a total of 10,227,670,521 vaccine doses have been administered around the world. However, in underdeveloped countries, most people must wait until 2023 for vaccination [4]; according to the World Health Organization (WHO)’s Africa Office, just over 3% of Africans are fully vaccinated against COVID-19 [5]. Despite the use of COVID-19 vaccines in many countries, the crisis is far from being resolved [6]. The ongoing evolution of SARS-CoV-2, the behavior of citizens, the decisions of governments in response to the pandemic and progress made in vaccine development and treatments will undoubtedly affect human life in the future. It is impossible to imagine when the world will return to “business as usual”, but what will remain is the lesson that this pandemic has provided to humankind, mostly in terms of prevention and behaviors. Nowadays, two years on from the beginning of the pandemic, new studies are aimed at post-acute COVID-19 syndrome (PCS), health-related quality of life (HRQoL) [7], “Long-COVID” [8], neurological complications [9,10], and so on. The structure, function, epidemiology and transmission of SARS-CoV-2 have been widely described in adults [11,12]. Fewer studies have reported on children, newborns [13] and pregnant women [14]. Newly arising SARS-CoV-2 variants are now a threat to global health and are creating COVID-19 surges in different countries. To date, there is limited knowledge about these emerging variants, which are now circulating globally [15]. At the time of writing, Europe is facing a fourth wave of COVID-19, while the third wave is not yet over, particularly due to low vaccination coverage. Increasingly transmissible variants (e.g., Delta and Omicron) are proliferating, and the risk of new and immunity-escaping variants is higher when populations are only partially vaccinated [16]. The Omicron variant is now dominant in at least 85 countries, raising concerns about its potentially high transmissibility [17]. It is yet been clearly understood whether vaccines will be effective against all of these variants [18]. Vaccine top-ups, rolled out in autumn 2021, might boost the immune system and block new coronavirus variants, but there are many complexities [19,20]. In most countries, contacts of confirmed COVID-19 cases are asked to quarantine for 14 days after exposure, to limit asymptomatic onward transmission even though, at the individual level, the 14-day quarantine may cause strain on mental health. There are recent studies aimed at reducing the length of quarantine for uninfected contacts [21], considering the difficulty to maintain the quarantine for a prolonged period. Another issue relating to the COVID-19 pandemic is also represented by increased alcohol consumption, which was observed during lockdown in people in quarantine and studying or working at home [22,23]. Besides the vaccination procedure, the primary examined therapies to treat COVID-19 patients include: antiviral regimens, such as remdesivir, ritonavir, lopinavir; antimalarials, such as chloroquine and hydroxychloroquine; antibacterials, such as azithromicin; immunomodulating drugs, such as corticosteroids and interleukin (IL) inhibitors; and other therapies including convalescent plasma infusion [24]. In particular, dexamethasone therapy plays a major role in the treatment of patients with COVID-19 pneumonia requiring supplemental oxygen or on mechanical ventilation [25]. Molnupiravir (MK-4482, EIDD-2801) is an oral antiviral drug working through the mechanism of “error catastrophe” against SARS-CoV-2 [26]. It increases the frequency of viral RNA mutations and impairs SARS-CoV-2 replication in animal models and in humans. Molnupiravir was authorized for COVID-19 treatment in the UK on 4 November 2021 [27]. Paxlovid^TM^ (nirmatrelvir  +  ritonavir), developed by Pfizer Inc., has been proposed by the U.S. Food and Drug Administration (FDA) for emergency use in infected adults at high risk of severe illness who are unvaccinated [28]. The advantage of these two pills over other antiviral compounds is that they are given at home [29]. If COVID-19 is diagnosed early, it has been suggested that the use of a combination of antivirals and an immunomodulator (e.g., a drug potentiating IFNα/β) may lower the risk of cytokine storm manifestation. When the disease becomes severe, the new combination should prioritize targeting the cytokine storm [30]. Lastly, vitamin D supplementation has been suggested for the prevention and/or treatment of COVID-19 [31], because of its well-known immunomodulatory role. Another aspect of the current COVID-19 outbreak is the rise of germaphobes that rarely leave the house without a bottle of hand sanitizer. However, it would be better to be more cautious and to think of possible consequences before occurrence. Indeed, there are broad-spectrum antibacterial agents used in sanitizers, such as triclosan and triclocarban, which are highly absorbed by the skin and at high doses have shown toxicity [32]. Moreover, some hand sanitizer products may contain unacceptable active ingredients (especially methanol) other than the recommended alcohols (ethanol or isopropyl alcohol), whose toxicity after dermal and respiratory exposure is known: if swallowed, it may lead to death. The COVID-19 pandemic is considered the largest methanol mass poisoning event around the world in recent decades [33]. Finally, because of the high antibiotic use in COVID-19 patients, there has been a rapid increase in antimicrobial resistance [34,35,36], which poses collateral damage in the COVID-19 pandemic. Overall, this review aims to provide a quick update of the drugs, vaccines, and therapeutic treatments used today for this devastating disease.

## 2. Diagnosis, Variants and Vaccines

### 2.1. Diagnosis

COVID-19 is generally diagnosed by reverse transcription (RT-PCR) or serological assays. Antibody testing, using a rapid immunochromatographic assay, is reliable in the diagnosis of SARS-CoV-2 infection; however, RT-PCR testing on oropharyngeal or nasopharyngeal swabs is an important and more sensitive tool with respect to antigenic testing even though several parameters can affect both assays. Recently, some concerns have been raised about the performance of RT-PCR testing [37], since patients who are ultimately proven to have COVID-19 may have a negative RT-PCR test, particularly early in the course of their disease, while false positive antibody tests are also a concern [38]. Thus, the combination of two methods, such as RT-PCR and the IgG assay, has been proposed to be more trustworthy [39]. In severe cases, clinicians need to rapidly and reliably diagnose COVID-19 for proper risk stratification, isolation strategies, and treatment decisions. Chest computed tomography (CT) has been demonstrated as being a high-performance test for diagnosing COVID-19 [40]. Recently, the combined use of chest X-ray images (CXI) and chest computer tomography images (CCTI) was proposed, by using the Fourier–Bessel series expansion-based decomposition (FBSED) method [41].

### 2.2. Mutations and Variants

As has been assessed, SARS-CoV-2 is genetically variable and easily adaptable to various hosts, including humans. Indeed, SARS-CoV-2 has accumulated around two mutations per genome each month, and several other variants have emerged [42]. The terminology of viral variation can be confusing, as the media and even scientific papers often use the terms variant, strain, and lineage interchangeably. Fortunately, the WHO is working on a systematic nomenclature that does not require a geographic reference, since viral variants can spread rapidly and globally [43]. Three nomenclature systems for naming and tracking SARS-CoV-2 genetic lineages are currently in use: Pango [44,45] (available online) [46], Global Initiative on Sharing All Influenza Data (GISAID) [47,48] and NextStrain [49,50]. The last two databases focus on broader phylogenetic ‘clades’ and incorporate criteria for minimum prevalence and persistence. Among the numerous variants, the WHO, CDC (USA) and European Centre for Disease Prevention (ECDC) (Europe) highlight some as significant ones and categorize them into two subgroups: variants of concern (VOCs) and variants of interest (VOIs), which are updated when required [51]. VOCs emerged as a greater threat to public health, with higher infectivity and transmissibility, and an increased virulence pattern. They can alter COVID-19’s clinical manifestations, decrease the efficacy of available vaccines and therapeutics and may obstruct the present ability of RT-PCR assays to detect the virus. The existence of different nomenclature systems means that the same variant has multiple names, often at the same time and without regard for the properties of VOIs and VOCs. By this time, a continuously enriched spectrum of SARS-CoV-2 genomic variants has been evidenced and categorized by the WHO using the Greek alphabet (Alpha to Omicron). In Table 1, the known SARS-CoV-2 variants, to date, are summarized.

### 2.3. Vaccines for COVID-19

By 1 September 2020, an enormous number of vaccines (more than 200) were in preclinical development, some of which had entered clinical trials [59]. The approved vaccines are summarized in Table 2. Four vaccines have been used in Italy: two mRNA vaccines, Pfizer-BioNTech mRNA BNT162b2 (Comirnaty) (USA) [60] and Moderna mRNA 1273 (USA) [61], and two recombinant viral vector adenoviruses, Astra-Zeneca ChAdOx1-S, belonging to Oxford University (UK) and Pomezia (Italy), and Janssen, belonging to Johnson & Johnson (USA) [62,63]. Firstly, two doses were needed to induce satisfactory immunity; however, the administration of third doses, called “booster doses”, of COVID-19 vaccines started in autumn 2021. The rapid development and deployment of vaccines against the SARS-CoV-2 virus has been an unprecedented scientific achievement. Thus, not all the side effects evidenced were predictable [64]. The viral vector-based vaccines are associated with a higher prevalence of systemic side effects (e.g., headache/fatigue), whereas the mRNA-based vaccines are associated with a higher prevalence of local side effects (e.g., injection site pain, muscle pain, malaise, chills, and joint pain). The vast majority of side effects resolve themselves within 1–3 days after vaccination [65]; however, severe side effects do also sometimes occur. The most frequent neurological side effects of SARS-CoV-2 vaccines are Guillain–Barre syndrome (GBS), venous sinus thrombosis (VST), and transverse myelitis, with VST particularly occurring after vaccination with a vector-based vaccine [66]. Cutaneous reactions after COVID-19 vaccination have also been commonly reported [67], as well as cases of erythema nodosum and pityriasis rosea after Oxford–AstraZeneca COVID-19 vaccine administration [68]. Moreover, reactivation of varicella zoster virus (VZV), as well as herpes zoster (HZ), after COVID-19 vaccination with both viral vectors and mRNA vaccines has been reported in a series of adult patients [69,70]. The new onset and exacerbation of psoriasis after COVID-19 vaccination have also been described with mRNA vaccines [71]. In children, the phenomena of encephalopathy and myocarditis following COVID-19 mRNA vaccination have been described [72]. In Italy, after the first dose, vaccine brands were mixed for people who received Johnson & Johnson and AstraZeneca vaccines, as these two vaccines were retired because of the severe side effects that occurred. Mixing adenoviral and mRNA vaccines has been demonstrated to be safe and tolerable [73]. Currently, only Pfizer and Moderna vaccines are administered in Italy. The recent emergence of the SARS-CoV-2 Omicron variant has become a global concern and the vaccine protection against this variant is still under debate [74].

## 3. Current Therapies 

**Figure 1:**
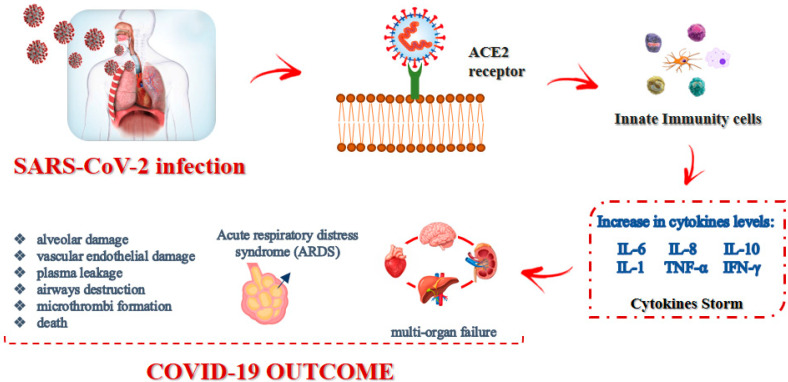
SARS-CoV-2 infection modality and effects.

Current therapies used for COVID-19 target the so-called “cytokine storm” (CS), also known as cytokine release syndrome (CRS) [77]. Indeed, COVID-19 is characterized by dramatically elevated levels of inflammatory cytokines, mainly IL-6, IL-8, IL-10, IL-1, tumor necrosis factor-α (TNF-α) and interferon-γ (IFN-γ), as depicted in Figure 1. These pro-inflammatory mediators can provoke systemic inflammatory response syndrome, resulting in acute respiratory distress syndrome (ARDS).

The pathological changes that generally occur in COVID-19 patients include diffuse alveolar damage, due to immunological injury and viral infection, as well as multiorgan failure, such as airway destruction, vascular endothelial damage, plasma leakage, and extensive microthrombi formation [78]. Pneumonia, frequently occurring in COVID-19 patients, may result as a direct consequence of the viral infection in the lung or arise because of secondary bacterial infections after the viral episode. Targeting the appropriate cytokine is one of the main objectives of current studies, and one of the first approaches to COVID-19 cytokine storm syndrome treatment was targeting the IL-6, since early during the pandemic, IL-6 concentrations were noted to be elevated. Unfortunately, most randomized controlled trials have not documented an improved survival rate using agents targeting IL-6; by contrast, targeting IL-1 was largely successful, as reported by a retrospective cohort study [79]. SARS-CoV-2, as with other viruses, triggers an antiviral response that relies on the immediate production of IFNβ in the host, which binds to its receptor (human interferon alpha-receptor, IFNAR) and triggers the production of IFNα. If the production of IFNα/β occurs immediately and is intense enough, the infection can be stopped, as often happens in asymptomatic or paucisymptomatic patients, especially in children. If the response is weak, the virus replicates, causing a second inflammatory/immune response, which may become explosive and potentially result in a cytokine storm and ARDS. 

**Figure 2:**
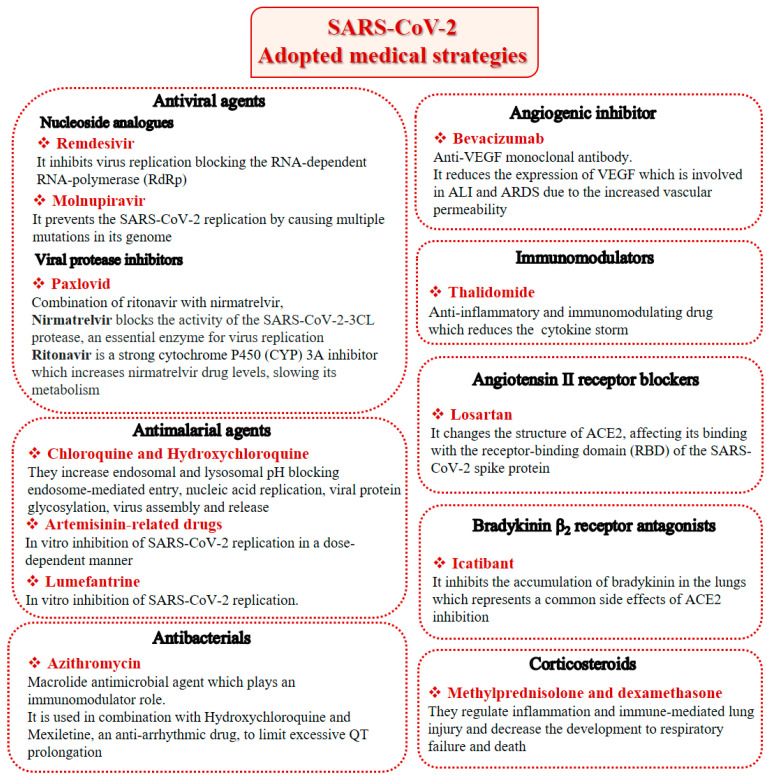
SARS-CoV-2 adopted medical strategies.

Beyond therapies targeting cytokines, other treatments have been extensively reviewed recently [80], namely antiviral agents, antimalarial agents, antibacterials, immunomodulators, angiotensin II receptor blockers, bradykinin B_2_ receptor antagonists, corticosteroids, anthelmintic, antiprotozoal, H_2_ blockers and anticoagulants. However, alternative and effective therapies to overcome the already known and/or the unforeseen possible manifestations of SARS-CoV-2 in the near future may be needed. The most important therapeutic drugs available for the treatment of COVID-19 are summarized in Figure 2. 

Remdesivir, a broad-spectrum antiviral agent able to inhibit SARS-CoV-2 replication, was the first available therapeutic drug for COVID-19. It is a small-molecule monophosphoramidate prodrug, as it is metabolized into the nucleoside triphosphate (active drug form) inside the cell through sequential reactions by ester-mediated hydrolysis, and an adenosine analog, blocking the RNA-dependent RNA-polymerase (RdRp) through its nucleoside component after the virus’ entry into the host cell. On 1 May 2020, on the basis of preliminary results from phase 3 trials, the Food and Drug Administration (FDA) issued an Emergency Use Authorization (EUA) allowing the use of Remdesivir for the treatment of suspected or laboratory-confirmed COVID-19 in adult and pediatric patients (over 12 years old and weighing at least 40 kg or more) hospitalized with severe disease [81]. An intravenous nucleotide prodrug of Remdesivir (Veklury^®^) is currently approved by the FDA for the treatment of SARS-CoV-2 infection, but its limitations and controversial efficacy make it difficult to be widely used in hospitalized patients [82].

Chloroquine and hydroxychloroquine are antimalarial drugs that inhibit the lysosomes’ vital functions by increasing the pH, which results in blocking endosome-mediated entry, and can interfere with nucleic acid replication, viral protein glycosylation, virus assembly, and release. In particular, Chloroquine possesses an immune-modulatory activity, which could enhance its antiviral effects, with some limitations because of the potential for overdosing, acute poisoning, and death. Even though hydroxychloroquine has been demonstrated to be much less toxic (by about 40%) [83], both are responsible for the onset of side effects, such as gastrointestinal disorders, headaches, retinopathy, and arrhythmia. Meanwhile, a large randomized clinical trial demonstrated that these drugs were not associated with mortality rate reduction, nor with recovery rate improvement [84]. After 15 June 2020, the FDA finally indicated that hydroxychloroquine and chloroquine were not beneficial for the treatment of COVID-19 [85].

Another old antimalarial drug derived from Chinese herbs, artemisinin, together with other artemisinin-related drugs, has been reported to have multiple pharmacological activities, including antiviral, anticancer and immune modulation ones [86]. Cao et al. (2020) [87] systematically explored the antiviral activities of several artemisinins against SARS-CoV-2 in vitro. Among them, artesunate and arteannuin B were found to be good anti-SARS-CoV-2 agents but, interestingly, the authors found that the antimalarial drug lumefantrine, even though it is structurally distant from artemisinins, could inhibit SARS-CoV-2 in vitro as well.

Molnupiravir (Lagevrio, Merck (Branchburg, NJ, USA)) is the first oral antiviral drug authorized for COVID-19 treatment in the UK starting from the 4 November 2021 for adults with a positive COVID test who have at least one risk factor for developing severe illness [27]. Molnupiravir prevents SARS-CoV-2 replication by causing multiple mutations in its genome, and demonstrated a significant benefit in reducing hospitalization or death in mild COVID-19 patients. However, its role in moderate to severe COVID-19 is still questionable [88]. 

Paxlovid (Pfizer (New York, NY, USA)) is a combination of ritonavir with nirmatrelvir, a SARS-CoV-2 protease inhibitor currently being assessed in phase 3 trials for its safety and efficacy in the treatment of non-hospitalized adult patients with COVID-19 who are not at risk of developing severe illness. Its clinical efficacy meant that it was able to reduce hospitalization by 80%, and it is also being explored as a post-exposure prophylaxis agent in patients previously exposed to SARS-CoV-2 [89].

Azithromycin is a macrolide antimicrobial agent which acts against a broad range of Gram-positive and Gram-negative bacteria and plays an immunomodulatory role and has been widely used for the treatment of COVID-19 patients with moderate to severe symptoms [90]. It is also used in combination with hydroxychloroquine, even though an increased risk of malignant arrhythmia and sudden cardiac death has been reported [91]. Mexiletine, a well-known anti-arrhythmic drug used in congenital long QT syndrome (LQTS) patients was proposed in association with hydroxychloroquine and azithromycin for the treatment of COVID-19 patients to limit excessive QT prolongation. In subclinical conditions, the model suggests that mexiletine may limit the deleterious effects of azithromycin and hydroxychloroquine [92,93].

As a monoclonal antibody, bevacizumab acts against the vascular endothelial growth factor (VEGF) and is usually indicated for cancer therapy; however, numerous investigations have supported the fundamental role of VEGF in acute lung injury (ALI) and ARDS, suggesting a potential role in the management of COVID-19 patients. Indeed, several clinical trials on the efficacy of bevacizumab in severe patients with COVID-19 are in different phases of study [94]. Again, thalidomide, an anti-inflammatory and immunomodulating drug that has demonstrated antiproliferative activity in cancer, has been proposed, since it significantly accelerated the negative conversion of SARS-CoV-2 and shortened the hospital stay length of affected patients [95]. It also reduced the requirement for mechanical ventilation in patients with critical COVID-19. Most importantly, the combination therapy of thalidomide with low-dose glucocorticoid was effective in improving the prognosis of patients with critical COVID-19.

Angiotensin II receptor (ACE2) antagonists, belonging to the class of sartans, are known antihypertensive agents and are now under study for COVID-19 treatment, starting from the early consideration that coronaviruses transfer their genetic material to the host cell, binding the ACE2 receptors. Losartan inhibits ACE2 binding to the AT1 receptor, thereby counteracting the physiological effects of angiotensin II [96]. ACE2 is also related to bradykinin, a potent inflammatory mediator, and hydrolyzes the active bradykinin metabolite [des-Arg973] BK (DABK). As a side effect, with the reduction in the amount of ACE2 in the body, COVID-19 activates the bradykinin system, leading to fluid extravasation and leukocyte recruitment to the lung, which persists in pulmonary edema subsequently. For this purpose, icatibant, a bradykinin B2 receptor inhibitor, has been introduced as a potential candidate for the treatment of COVID-19 [97].

Finally, the use of corticosteroids in patients with COVID-19 is still controversial, since the World Health Organization (WHO) and the Centers for Disease Control and Prevention (CDC) generally advised the use of glucocorticoids against COVID-19 pneumonia, unless in specific comorbid clinical conditions, e.g., exacerbation of chronic obstructive pulmonary disease. Indeed, it seems that clinical phenotypes are associated with a differential response: for critical COVID-19 patients, corticosteroid therapy was associated with a decrease in 28-day mortality, but the use of corticosteroids showed significant survival benefits in patients with the hyperinflammatory phenotype [98]. In hospitalized hypoxic COVID-19 patients, methylprednisolone demonstrated better results compared to dexamethasone [99]. Structures for described compounds are reported in Table 3. The fight against COVID-19 is not finished yet, and new research focused on investigations of further drugs use/association in the management of the most severe effects is needed. 

## 4. Alternative Therapies

Several studies on phytonutrients, dietary supplements and nutraceuticals for the treatment and prevention of COVID-19 have been reported [100]. Such products include vitamin C, vitamin D, polyphenols, omega 3 polyunsaturated fatty acids, probiotics, and zinc, all of which are currently under clinical investigation [101]. Several studies on the potential role of vitamin D in COVID-19 in adults, older people and children are ongoing [102,103]. A recent study reported that the administration of high-dose intravenous vitamin C (HDIVC) is beneficial for critically ill COVID-19 [104]. However, other authors found no significantly positive outcomes in patients with severe COVID-19 disease treated with HDIVC [105]. Polyphenols, such as epigallocatechin gallate (EGCG) and gallocatechingallate (GCG), which are common constituents of green tea, quercetin (found abundantly in apples) and hesperetin (in citric foods) are currently under study [106,107,108]. Spices such as turmeric, thyme, rosemary, and garlic are also under investigation for the prevention and treatment of COVID-19 [109]. Five selected flavonoids, namely curcumin, kaempferol, quercetin, apigenin and monolaurin, have been critically examined for use as adjuvant therapeutic agents against viral infections, including SARS-CoV-2 [110]. Quercetin was suggested as a reasonably potent inhibitor of SARS-CoV-2 3CLpro protease [111]. Curcumin supplementation was demonstrated to decrease common symptoms, the duration of hospitalization and deaths, through the partial restoration of pro-/anti-inflammatory balance [112]. Studies on *Mentha piperita* demonstrated the existence of active compounds in mint leaves (*M. piperita* L.), including rutin, hesperidin, and isorhoifolin [113,114]. Currently, a double-blinded clinical trial (participant, researcher) on pomegranate juice is underway [115,116]. Probiotics, such as lactobacillus and bifidobacterium, have numerous benefits such as balancing the composition of human gut microflora, strengthening gut barrier function, and protective immune responses. They may serve as lead prophylactic and immune-boosting probiotics in COVID-19 management [117]. Recently, melatonin, the so-called ’hormone of darkness’, due to its synthesis at night-time, has attracted the interest of infectious diseases specialists and epidemiologists for its adjuvant role in treating patients with COVID-19 [118]. It has been recently given as part of a US Food and Drug Administration emergency authorized cocktail, REGEN-COV2, for the management of COVID-19 progression [119]. 

Staying at home for quarantine and consequently being largely inactive is associated with unintended consequences. These measures can actually raise the infection risk and exacerbate poor health conditions involving the immune system. Physical activity and yoga may represent a feasible way of improving health, particularly physical and mental health [120,121]. Irisin, a muscle-contraction-induced immunomodulatory myokine that is released from myocytes during physical activity, has been proposed as a possible strategy to tackle COVID-19 [122,123], even though, to date, only a few articles have reported the use of irisin in COVID-19 [124]. Finally, the combination of active natural products with approved antiviral drugs has recently been suggested as an alternative to improve the biological effect and/or produce a synergistic outcome as supportive agents to minimize clinical symptoms [125]. 

## 5. Repositioning Drugs

The discovery of new drugs is a long, costly, and rigorous scientific process; therefore, a more effective approach could be represented by the search for effective anti-SARS-CoV-2 therapies from existing drug databases. Thus, drug repositioning, also known as drug repurposing, has emerged as a successful strategy for uncovering the potential of drugs in the management of diverse diseases. Since it concerns the investigation of existing drugs for new therapeutic target indications, the major benefits are represented by the reduced costs and expedited approval procedures. The urgency to find drugs fighting COVID-19 has tremendously pushed this kind of research [126,127]; indeed, in the last year, computational drug repositioning represented one of the most rapid and winning strategies applied for discovering SARS-CoV-2 drugs [128]. A total of 1553 FDA-approved drugs, as well as another 7012 investigational or off-market drugs in DrugBank, were proposed [129]. As an emblematic example, the first treatment available for COVID-19, remdesivir, was proposed as a repositioned drug [130]. Virtual screening studies showed that broad-spectrum antiviral agents (BSAAs) revealed potential activity against the SARS-CoV-2 spike glycoprotein, RdRp, the main protease (Mpro) and the helicase enzyme of SARS-CoV-2. In particular, imatinib (a tyrosine kinase inhibitor), suramin (an anti-parasitic), glycyrrhizin (an anti-inflammatory) and bromocriptine (a dopamine agonist) revealed good affinity towards multiple viral targets [131]. 

Recently, in silico screening followed by wet-lab validation indicated a poly-ADP-ribose polymerase 1 (PARP1) inhibitor, CVL218, currently in phase I clinical trial, for repositioning to treat COVID-19. CVL218 was able to inhibit SARS-CoV-2 replication without exhibiting any obvious cytopathic effects; moreover, CVL218 can interact with the nucleocapsid protein of SARS-CoV-2 and suppress the LPS-induced production of several inflammatory cytokines that are highly relevant to the prevention of the immunopathology induced by SARS-CoV-2 infection [132]. Several other compounds, endowed with different biological activities, have been proposed for repositioning [133], such as ivermectin (IVM), an FDA-approved antiparasitic drug for onchocerciasis and strongyloidiasis [134], fibrates, diarylureas [135,136], Schiff bases [137,138,139] and transition metal complexes [140,141]. Moreover, a recent article indicated the use of repositioning microbial biotechnology for studies against COVID-19, particularly for flavonoids. The idea is that the adoption of new systems and synthetic biological tools could provide an optimal framework for the guided and targeted expansion of flavonoid chemical diversity [142]. Finally, repositioning drugs may be used to counteract the side effects of currently used drugs, such as drug-induced LQTS. The common cause of LQTS induced by drugs is due to the impairing of human ether-ago-go-related gene (hERG) channels. For instance, among COVID-19 treatment drugs, chloroquine and hydroxychloroquine were demonstrated to block hERG potassium channels, whereas azithromycin and remdesivir did not [143]. Thus, the addition of hERG potassium channels inhibitors, such as lubeluzole, to the conventional therapies may represent an interesting strategy to overcome LQTS [144,145].

## 6. Recent Preclinical Studies

A recent study was focused on aminothiol cysteamine, a human existing drug, and its disulfide product of oxidation, cystamine, which have anti-infective properties targeting viruses, bacteria, and parasites. The antiviral effects against SARS-CoV-2 were evaluated using different in vitro viral-infected cell-based assays. Moreover, the ability of cysteamine to modulate the SARS-CoV-2-specific immune response was studied in vitro in blood samples from COVID-19 patients. Both compounds were shown to decrease SARS-CoV-2-induced cytopathic effects (CPE) in Vero E6 cells. Cysteamine and cystamine significantly decreased the viral production in Vero E6 and Calu-3 cells, and their antiviral action did not depend on the treatment time with respect to SARS-CoV-2 infection. Additionally, they showed an anti-inflammatory effect by lowering the SARS-CoV-2 specific IFN-γ production in vitro, using blood samples from COVID-19 patients [146]. 

Recent studies addressed the most feared novel Omicron variant. Nirmatrelvir, molnupiravir, and remdesivir were tested in vitro against a panel of SARS-CoV-2 variants in live-virus antiviral assays. It was demonstrated that nirmatrelvir, as well as other clinically relevant antivirals, maintains its activity against all variants tested, including Omicron [147], confirming what was previously reported [148,149]. The isolation of the Omicron variant was reported for the first time by Yadav et al. (2022) [150] by using an in vivo followed by in vitro method of SARS-CoV-2 virus culture; more specifically, it was obtained from infected Syrian hamsters and the subsequent passage into Vero CCL-81 cells, since the Omicron variant’s isolation in Vero CCL-81 from the clinical specimens of COVID-19 cases failed. Recent in vitro studies have been carried out to evaluate therapeutic antibodies against a SARS-CoV-2 Omicron B.1.1.529 isolate. The authors found that a dose of 500 mg of sotrovimab retained a significant level of neutralizing activity against the Omicron compared to previous variants. The activity of evusheld 300 mg represented about 10% of the activity of sotrovimab 500 mg [151].

Finally, a high-throughput screening test was performed by some authors to explore the in vitro antiviral activity of itraconazole and its metabolite hydro-itraconazole, already known for the feline coronavirus and influenza A virus [152]. The positive pharmacological and pharmacokinetic properties led the authors to adopt a hamster SARS-CoV-2 infection model to study the possible activity of itraconazole in acute infection and viral transmission models. However, the results were disappointing, given that the administration of itraconazole in hamsters was unable to reduce the viral RNA load in the lungs, ileum or stools and did not mitigate pulmonary inflammation, despite sufficient exposure.

## 7. Recent Clinical Studies

A recent interesting study described the production of the first chicken egg yolk-derived anti-index SARS-CoV-2 receptor-binding domain (RBD) immunoglobulin Y (IgY) polyclonal antibodies as an intranasal drop product for humans, with equal in vitro activity against all VOCs. The major advantages are the ease and fast production and the low cost; moreover, the antibodies were developed as nasal drops, in order to capture the virus directly on the nasal mucosa. In this double-blinded, randomized, placebo-controlled phase 1 study, single-ascending and multiple doses of anti-SARS-CoV-2 RBD IgY were administered intranasally for 14 days in 48 healthy participants, demonstrated an excellent safety and tolerability profile, and the absence of systemic absorption of topically administered IgY. Moreover, no evidence of a systemic inflammatory immune response triggered by the topical treatment with anti-SARS-CoV-2 RBD IgY in humans was observed [153]. Recently, the valuable beneficial effects of beta glucans, derived from two strains AFO-202 and N-163 of a black yeast *Aureobasidium pullulans*, were described. They were evaluated on the biomarkers for cytokine storm and coagulopathy in COVID-19 patients, in a randomized pilot clinical study, where the supplementation with these beta glucans helped to maintain the major biomarkers of clinical severity and mortality of COVID-19, such as IL-6, D-Dimer, neutrophil to lymphocyte ratio (NLR) over 15 and 30 days, compared to those who underwent standard care alone [154]. Another recent pilot study, an open label, randomized clinical trial, investigated the potential benefit of calcitriol therapy given to 50 patients hospitalized with COVID-19 indicating an improvement in oxygenation, suggesting the need for a larger randomized trial [155]. Studies in IVM are debated. A randomized, double-blinded, multicenter, phase II, dose-finding, proof-of-concept clinical trial showed that high-dose IVM led to a not significant reduction in viral load when compared to placebo. Whether this drug might have clinical efficacy at lower doses remains debated. The recommendation of the WHO suggests that it is currently advisable to refrain from administrating ivermectin for the treatment of COVID-19 outside clinical trials [156]. These studies are summarized in Table 4.

## 8. Conclusions

As a matter of fact, the pandemic is still far from over. In this paper, we summarized the most important features regarding COVID-19, the human respiratory illness caused by the virus SARS-CoV-2. Studies concerning diagnosis, treatment and vaccines for this disease have been addressed, together with the worth of rapid diagnostics, which is fundamental. This aspect is only partially satisfied by the available tests, in our opinion, considering that the viral genomic detection has been exclusively based on molecular tests, mainly RT-PCR, and that the more rapid antibody (serology) tests, necessary for a wider immune screening, cannot confirm with certainty the virus’ presence. The COVID-19 pandemic prompted scientists from around the world to design anti-SARS-CoV-2 vaccines and try several therapies, most of them obtained by exploiting the approach of drug repositioning, a field of drug research whose importance has increased in recent years, due to several advantages, such as the possibility to shorten the clinical trials and also the life extension of old drugs by finding a new therapeutic target. In spite of these efforts, proper and effective therapies for COVID-19 have still not been discovered, mostly because of the presence of different forms and variants of this disease. However, among the therapies currently used, antibiotics are at the forefront, and, for this reason, particular attention must be paid to the emergence of multidrug-resistant bacteria, a very important issue that must be taken into account to avoid the so-called “jumping out of the frying pan into the fire”. Appropriate prescriptions, optimized antibiotic use and aggressive infection control may help to prevent the occurrence of multidrug resistance (MDR). Additionally, efficient and early diagnosis, combined with effective treatments, might significantly lessen the burden of other pandemic waves. 

There is also the need to develop additional safe, effective, easy-to-produce, and inexpensive preventive tools to reduce the risk of acquiring SARS-CoV-2 infection. New preclinical studies, i.e., those with intranasal delivery of anti-SARS-CoV-2 IgY, have been suggested to provide passive immunization, including for use as an add-on to personal protective equipment and other preventive measures for the general population until vaccination, which is hopefully highly effective against prevalent variants, becomes available worldwide or herd immunity is achieved. This IgY may also provide short-term protection, in addition to vaccines, in less well-ventilated environments, including trains, airplanes, lecture halls, etc. To conclude, it is also necessary to emphasize that the recently described VOC Omicron (B.1.1.529) variant has rapidly spread worldwide and is now responsible for the majority of COVID-19 cases in many countries, in which less severe clinical diseases in humans are registered. Finally, it is desirable that, although many knowledge gaps exist about the epidemiology, clinical severity, and disease course of the Omicron variant, its high transmissibility could potentially contribute to the achievement of the desired herd immunity.

## Figures and Tables

**Table 1:** Variants of SARS-CoV-2.

Variant Name (Pango Lineage)	GISAID Database	Nextstrain Database	WHO Names		Variant Name (Pango Sublineage)	WHO Designation	Date of Designation
B.1.525	G/484K.V3	20A/S:484K	Eta	η	–	VOI	UK/Nigeria December 2020
B.1.526	GH	20C/S:484K	Iota	ι	–	VOI	New York, NY, (USA) November 2020
–	–	20C	–	–	B.1.526.1	VOI	USA October 2020
B.1.617	G/452R.V3	20A					
		20A/S:154K	Kappa	κ	B.1.617.1	VOI	India December 2020
–	G/452R.V3	20A/S:478K	Delta	δ	B.1.617.2	VOC	India October 2020
–	–	20A	–	–	B.1.617.3	VOI	India October 2020
C.37	–	–	Lambda	λ	–	VOI	Lima November 2020
B.1.621	–	–	Mu	μ	–	VOI	Colombia January 2021
P.2	GR	20B/S:484K	Zeta	ζ	B.1.1.28.2	VOI	Rio de Janeiro (Brazil) April 2020
P.3	GR	20B/S:265C	Theta	θ	B.1.1.28.3	VOI	Japan/Philippines, January 2021
B.1.616	–	20C	–	–	–	VOI	France January 2021
B1.1.7	GRY (formerly GR/501Y.V1)	20I/S:501Y.V1	Alpha	α	–	VOC	South-East England (UK), September 2020
B1.351	GH/501Y.V2	20H/501Y.V2	Beta	β	–	VOC	South Africa, May 2020
P.1	GR/501Y.V3	20J/S:501Y.V3	Gamma	γ	B.1.1.28.1 or P.1	VOC	Japan/Amazonas (Brazil), November 2020
B.1.427	GH/452R.V1	20C/S:452R	Epsilon	ε	–	VOI ^a^	Southern California (USA) December 2020
B.1.429	GH/452R.V1	20C/S:452R	Formerly Epsilon	Formerly ε	–	VOI ^a^	Southern California, CA, (USA)
B.1.620	–	–	–	–	B1.177	VOC	Lithuania April 2021
–	–	–	–		B.1.258D	Other	Czech Republic/Slovakia
–	–	–	–		B.1.1.298	Other	Denmark
BA.1/BA.2	–	–	Omicron	o	B.1.1.529	VOC	South Africa (early Nov. 2021)

^a^ VOC (CDC, USA). Data were adapted from refs. [52,53,54,55,56,57,58].

**Table 2:** COVID-19 vaccines.

Manufacturer/WHO EUL Holder	Name of Vaccine	Platform	NRA of Record	Recommendation Issued
Pfizer Biontech	BNT162b2/COMIRNATY^®^ Tozinameran (INN)	Nucleoside modified mRNA	EMA FDA	31 December 20 16 July 21
Astra Zeneca	AZD1222 VAXZEVRIA	Recombinant ChAdOx1 adenoviral vector encoding the Spike protein antigen of the SARS-CoV-2	EMA MFDS KOREA Japan MHLW PMDA Australian TGA	15 April 21 15 February 21 9 July 21
Serum Institute of India	Covishield^TM^ (ChAdOx1_nCoV-19)	Recombinant ChAdOx1 adenoviral vector encoding the Spike protein antigen of the SARS-CoV-2	DCGI	15 February 21
Janssen	Ad26.COV2.S	Recombinant, replication-incompetent adenovirus type 26 (Ad26) vectored vaccine encoding the SARS-CoV-2 Spike (S) protein	EMA	12 March 21
Moderna	mRNA-1273 SPIKEVAX	mRNA-based vaccine encapsulated in lipid nanoparticle (LNP)	EMA MFDS FDA	30 April 21 23 December 21 6 August 21
Sinopharm (Beijing, Wuhan)/BIBP	SARS-CoV-2 Vaccine (Vero Cell), Inactivated (lnCoV)	Inactivated, produced in Vero cells	NMPA	7 May 21
Sinovac	SARS-CoV-2 Vaccine (Vero Cell), Inactivated (lnCoV) CoronaVac	Inactivated, produced in Vero cells	NMPA	1 June 21
The Gamaleya National Center of Epidemiology and Microbiology	Sputnik V	Human adenovirus vector-based COVID-19 vaccine	Russian NRA	11 August 20
CanSinoBIO- Beijing Institute of Biotechnology	Ad5-nCoV	Inactivated, produced in Vero cells	NMPA	–
Bharat Biotech, India	SARS-CoV-2 Vaccine, Inactivated (Vero Cell)/COVAXIN^®^	Whole-virion inactivated Vero cell	DCGI/CDSCO	3 November 21
Novavax	NVX-CoV2373/Covovax NUVAXOVID™	Recombinant nanoparticle prefusion spike protein formulated with Matrix-M™ adjuvant	EMA	21 December 21
CureVac	Zorecimeran (INN) concentrate and solvent for dispersion for injection; Company code: CVnCoV/CV07050101	mRNA-based vaccine encapsulated in lipid nanoparticle (LNP)	EMA	–
Sanofi Pasteur	CoV2 preS dTM-AS03 vaccine	Recombinant, adjuvanted	EMA	–

Adapted from refs. [75,76] Abbreviations: NRA, National Regulatory Agency; EMA, European Medicines Agency; FDA, Food and Drug Administration; MFDS, Ministry of Food and Drug Safety; MHLW, Ministry of Health, Labour, and Welfare; PMDA, Pharmaceuticals and Medical Devices Agency; TGA, Therapeutic Goods Administration; DCGI, Drugs Controller General of India; NMPA, National Medical Products Administration; CDSCO, Central Drugs Standard Control Organization.

**Table 3:** Structure of current drugs used for COVID-19 treatment.

Structure	Name	Class	Ref.
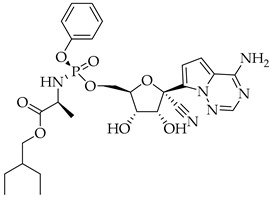	Remdesivir	Antiviral Nucleoside analogue	[81,82]
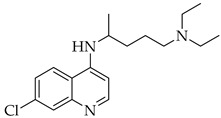	Chroloquine	Antimalarial	[83,84,85]
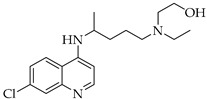	Hydroxychloroquine	Antimalarial	[83,84,85]
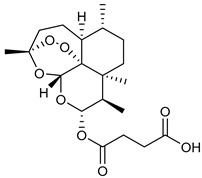	Artesunate	Antimalarial	[86,87]
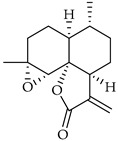	Arteannuin B	Antimalarial	[86,87]
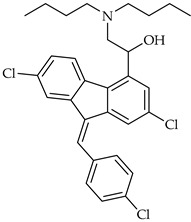	Lumefantrine	Antimalarial	[86,87]
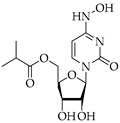	Molnupiravir	Antiviral Nucleoside analogue	[27,88]
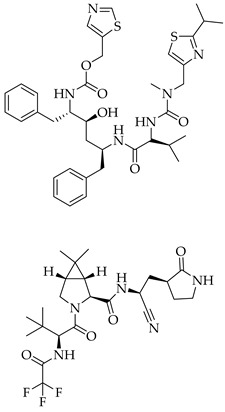	Paxlovid (ritonavir + nirmatrelvir)	Antiviral Viral protease inhibitor	[89]
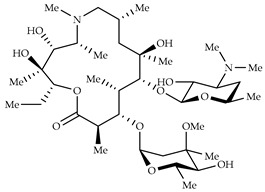	Azithromycin	Antimicrobial	[91]
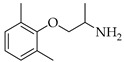	Mexiletine	Antiarrhythmic	[92,93]
Monoclonal antibody	Bevacizumab	Angiogenesis inhibitor	[94]
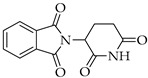	Thalidomide	Immunomodulator	[95]
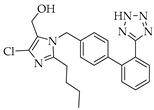	Losartan	Angiotensin II receptor blockers	[96]
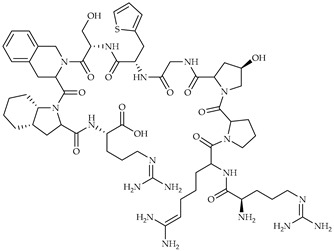	Icatibant	Bradykinin B2 receptor antagonist	[97]
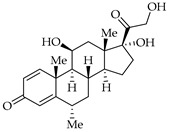	Methylprednisolone	Corticosteroid	[98,99]
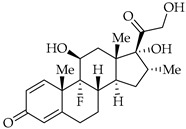	Dexamethasone	Corticosteroid	[98,99]

**Table 4:** Recent clinical studies.

Type of Clinical Studies	Treatments	Participants	Administration	Results
Double-blind, randomized, placebo-controlled phase 1 study	Chicken egg yolk-derived anti-index SARS- CoV-2 RBD IgY polyclonal antibodies as an intranasal drop product	48 healthy adults	Intranasally single-ascending doses of 2, 4, and 8 mg for 14 days	Excellent safety and tolerability profile and absence of systemic absorption
Randomized multiple-arm pilot clinical study	Beta glucans derived from two strains AFO-202 and N-163 of a black yeast *Aureobasidium pullulans*	24 RT-PCR positive COVID-19 patients	Additional supplementation for 30 days Group 1 control Group 2: AFO-202 beta glucan Group 3: a combination of AFO-202 and N-163 beta glucans	Significant control of IL6, D-Dimer and NLR, a significant increase in LCR, LeCR and marginal control of ESR in COVID-19 patients
Open label, randomized clinical trial	Calcitriol for the treatment of COVID-19	50 patients hospitalized with COVID-19	0.5 μg daily for 14 days	Improvement in oxygenation among hospitalized
Randomized, double-blind, multicenter, phase II, dose-finding, proof-of-concept clinical trial	Ivermectin for the treatment of COVID-19	89 adults recently diagnosed with asymptomatic/oligosymptomatic SARS-CoV-2 infection	(A) placebo (B) single-dose 600 μg/kg plus placebo for 5 days (C) single-dose 1200 μg/kg for 5 days	No significant reduction in viral load between ivermectin and placebo groups

## Data Availability

Not applicable.

## Abbreviations

ACE2	Angiotensin II receptor
ALI	Acute lung injury
ARDS	Acute respiratory distress syndrome
BSAAs	Broad-spectrum antiviral agents
CCTI	Chest computer tomography image
CDC	Centers for Disease Control and Prevention
CDSCO	Central Drugs Standard Control Organization
COVID-19	Coronavirus Disease 2019
CPE	Cytopathic effects
CRS	Cytokine release syndrome
CS	“Cytokine storm”
CT	Computed tomography
CXI	Chest X-ray image
DABK	Bradykinin metabolite [des-Arg973] BK
DCGI	Drugs Controller General of India
EGCG	Epigallocatechin gallate
ECDC	European Centre for Disease Prevention
EMA	European Medicines Agency
EUA	Emergency Use Authorization
FBSED	Fourier-Bessel series expansion-based decomposition
FDA	Food and Drug Administration
GBS	Guillain-Barre syndrome
GCG	Gallocatechingallate
GISAID	Global Initiative on Sharing All Influenza Data
HDIVC	High-dose intravenous vitamin C
hERG	Human ether-ago-go-related gene
HRQoL	Health-related quality of life
HZ	Herpes zoster
IFNAR	Human interferon alpha-receptor
IFN	Interferon
IgY	Immunoglobulin Y
IL	Interleukin
IVM	Ivermectin
LQTS	Long QT syndrome
MDR	Multidrug resistance
MFDS	Ministry of Food and Drug Safety
MHLW	Ministry of Health, Labour, and Welfare
Mpro	Main protease
NLR	Neutrophil to lymphocyte ratio
NMPA	National Medical Products Administration
NRA	National Regulatory Agency
PARP1	Poly-ADP-ribose polymerase 1
PCS	Post-acute COVID-19 syndrome
PMDA	Pharmaceuticals and Medical Devices Agency
RBD	Receptor-binding domain
RdRp	RNA-dependent RNA polymerase
RT-PCR	Reverse transcriptase-polymerase chain reaction
SARS-CoV-2	Severe acute respiratory syndrome coronavirus 2
TGA	Therapeutic Goods Administration
TNF-α	Tumor necrosis factor-α
VEGF	Vascular endothelial growth factor
VOCs	Variants of concern
VOIs	Variants of interest
VST	Venous sinus thrombosis
VZV	Varicella zoster virus
WHO	World Health Organization
